# Risk of asthma in individuals with eosinophilic esophagitis: Population‐based cohort study with sibling analyses

**DOI:** 10.1002/clt2.70068

**Published:** 2025-05-31

**Authors:** Niki Mitselou, Amiko Uchida, Bjorn Roelstraete, Erik Melén, John J. Garber, Jonas F. Ludvigsson

**Affiliations:** ^1^ Department of Pediatrics Faculty of Medicine and Health Örebro University Örebro Sweden; ^2^ Division of Gastroenterology, Hepatology & Nutrition University of Utah School of Medicine Salt Lake City Utah USA; ^3^ Department of Medical Epidemiology and Biostatistics Karolinska Institutet Stockholm Sweden; ^4^ Department of Clinical Science and Education Södersjukhuset Karolinska Institutet Stockholm Sweden; ^5^ Gastrointestinal Unit Massachusetts General Hospital Harvard Medical School Boston Massachusetts USA; ^6^ Department of Medicine Columbia University College of Physicians and Surgeons New York New York USA

**Keywords:** asthma, cohort, comorbidity, eosinophilic esophagitis, epidemiology

## Abstract

**Introduction:**

There are limited data on the relationship between eosinophilic esophagitis (EoE) and asthma. We aimed to assess the risk of asthma in EoE patients compared with matched controls and siblings.

**Methods:**

Through the ESPRESSO study, a Swedish nationwide population‐based histopathology cohort, we identified EoE patients diagnosed between 1989 and 2017 (*n* = 1146) and up to 5 age‐ and sex‐matched controls (*n* = 5022). Cox regression generated hazard ratios (HRs) for developing asthma. We compared EoE patients with sibling controls.

**Results:**

The median age at EoE diagnosis was 42 years. During a median follow‐up of 3.8 years, 140 EoE patients (28.1/1000 person‐years) and 174 controls (7.2/1000 person‐years) developed asthma (HR = 3.96; 95% confidence interval [CI] = 3.16–4.96, *p* < 0.001). An increased risk of asthma was seen in the first 10 years after EoE diagnosis but not thereafter. EoE patients diagnosed in childhood or young adulthood were at a particularly high risk of asthma (HR = 4.74; 95% CI = 2.93–7.67, *p* < 0.001 and HR = 5.84; 95% CI = 3.68–9.29, *p* < 0.001, respectively). Compared with their non‐EoE siblings, EoE patients were at a 5‐fold increased risk of asthma (HR = 4.97; 95% CI = 3.13–7.92, *p* < 0.001).

**Conclusion:**

EoE patients are at an increased risk of asthma compared with the general population, which is unlikely to be entirely explained through unmeasured intrafamilial factors given that the positive association remained in sibling analyses. Physicians caring for EoE should have a high awareness of concomitant asthma.

## 1 INTRODUCTION

Eosinophilic esophagitis (EoE) is an increasingly recognized chronic immune‐mediated disorder that is linked with a significant impairment in quality of life.^1^ Usually diagnosed in adolescence and young adulthood,^1^ EoE is clinically characterized by esophageal dysfunction and histologically by eosinophil‐predominant inflammation.^2^ Considering the life‐long nature of EoE, it is essential to examine and understand its complications and comorbidities.

There is evidence that EoE is associated with other atopic diseases,^2^, ^3^, ^4^, ^5^ and one review suggested that EoE and asthma frequently co‐exist in both children and adult patients.^6^ A meta‐analysis of 21 studies on 53,592 individuals with EoE and 54,759 controls reported a 3‐fold increased risk of asthma among both children and adults with EoE; odds ratio (OR) 3.06; 95% confidence interval (CI) = 2.01–4.66.^4^ In a US study involving 381 children with EoE, asthma or other allergic conditions such as allergic rhinitis and/or eczema were detected in 53% of the study participants.^7^ Similarly, Chehade et al. reported concomitant asthma in 45.4% in their cohort of 705 both pediatric and adult EoE patients.^8^ Though still evolving and, to date, poorly understood, the link between EoE and asthma seems to be bidirectional.^3^ EoE and asthma are both characterized by Th2‐driven inflammation, involving cytokines such as IL‐4, IL‐5, and IL‐13, with overlapping pathophysiology and treatment strategies.^6^

Despite the increasing evidence of an existing EoE‐asthma relationship, data are still limited as rigorous EoE epidemiologic studies in the context of asthma comorbidity, in particular new‐onset asthma, are lacking. The criteria for defining a diagnosis of asthma or other atopic manifestations vary widely across prior studies, mostly based on patient‐ or parental‐reported history rather than physician‐assigned diagnoses while some studies report no details on the diagnostic method.^4^ All in all, a better understanding of the relationship between EoE and asthma, and exploring shared pathophysiologic mechanisms may lead to novel diagnostic and management strategies. We therefore aimed to perform a nationwide study of biopsy‐verified EoE patients, up to 5 matched reference individuals randomly selected from the Swedish general population, and unaffected siblings of EoE patients to test the hypothesis that EoE is associated with an increased risk of asthma.

## 2 METHODS

### 2.1 Study design and population

This nationwide cohort study used prospectively collected data from Swedish health and welfare registers. Assigned at birth to all residents in Sweden, the personal identity number enables the linkage of data between different national registers for research purposes.^9^ In this study, we linked data from the Total Population Register (TPR),^10^ the National Patient Register (NPR),^11^ the Prescribed Drug Register (PDR),^12^ and the nationwide Epidemiology Strengthened by Histopathology Reports in Sweden (ESPRESSO) histopathology cohort.^13^

The ESPRESSO cohort includes data from over 6 million gastrointestinal biopsies registered according to the SNOMED‐CT System (Systematized Nomenclature of Medicine Clinical Terms) and collected from 1965 to 2017 from all 28 pathology departments in Sweden. The study population consisted of all individuals with biopsy‐verified EoE from 1989 to 2017 identified by searching the ESPRESSO cohort for SNOMED codes T62 (esophagus), and M47150 (eosinophilia). We restricted our analyses to EoE individuals without a prior diagnosis of asthma (*N* = 1146). A validation study previously conducted by our research group found that the positive predictive value (PPV) for a SNOMED record of EoE is 89% for EoE diagnosis.^14^ Food impaction was reported in 58% of the patients while dysphagia was seen in 70%.^14^ The *follow‐up* time in this study was from 1989 to 2019. Exclusion criteria included emigration during the study period or asthma diagnosis before the biopsy date/matching date.

### 2.2 Reference individuals (healthy comparators)

For each patient with EoE, up to 5 reference individuals (*n* = 5022) were identified and matched for sex, age, county, and calendar year of diagnosis from the Swedish TPR.^10^ At the matching date, none of the reference individuals had EoE or asthma. If any comparators developed EoE during the study period, their follow‐up was censored.

### 2.3 Siblings

**FIGURE 1:**
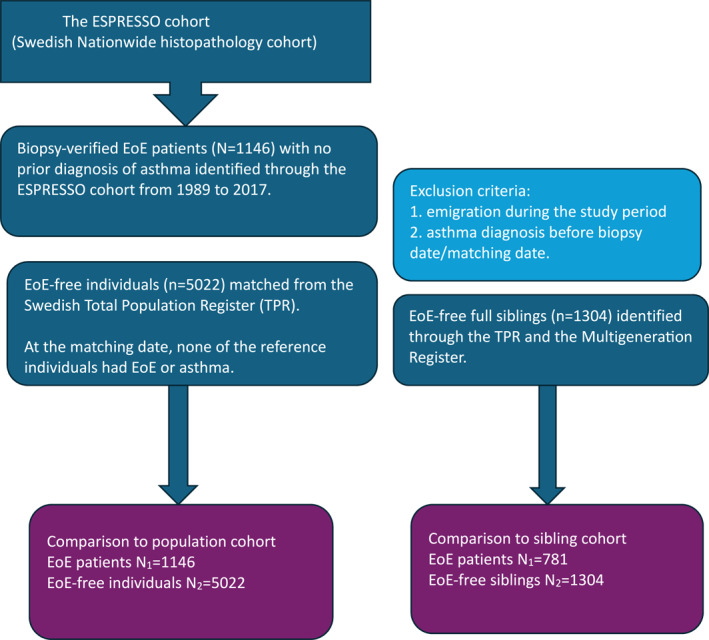
Flowchart for the matched cohort study. EoE, eosinophilic esophagitis; ESPRESSO, the Epidemiology Strengthened by histoPathology Reports in Sweden cohort.

Secondary comparisons using sibling analyses were performed to reduce the influence of shared genetics and some early environmental exposures, thus minimizing residual confounding. Unaffected full siblings (*n* = 1304) of 781 individuals with EoE were identified through the TPR and Multigeneration Register (Figure 1). Sibling data were available for all individuals born after 1932 and who were registered as residents of Sweden after 1961.

### 2.4 Covariates

In a separate analysis, we stratified by educational level: compulsory school (0–9 years); upper secondary (10–12 years); college or university (≥13 years). Individuals without data on educational status were categorized as “missing.” Further, to take autoimmunity into account, we calculated the risk of asthma in EoE individuals with other concomitant autoimmune diseases (see Supporting Information S1: Appendix).

Through the Swedish PDR,^12^ we obtained data on dispensed prescriptions for the following proton pump inhibitors (PPIs): omeprazole, pantoprazole, lansoprazole, rabeprazole, esomeprazole, dexlansoprazole, and dexrabeprazole (corresponding Anatomic Therapeutic Chemical (ATC) codes are listed in Table S1).

### 2.5 Outcome

The NPR includes inpatient and, since 2001, hospital‐based outpatient data. The register uses the Swedish International Classification of Disease (ICD) coding system to code diagnostic data, while the PPV for most chronic disorders ranges from 85% to 95%.^11^ The PDR is a nationwide database where all prescribed drugs dispensed at pharmacies were registered July 2005 onward.^12^ In this study, asthma was defined as having a relevant ICD‐9 (493) or ICD‐10 (J45 and 46) code in the NPR and/or as having a record of at least two dispensed prescriptions of asthma medications in the PDR: inhaled corticosteroids (ICS), β‐agonists, combination products or leukotriene receptor antagonists, using the following corresponding ATC classification codes: R03BA, R03AC, R03AK, and R03DC, respectively. To increase sensitivity, we used data from both registers to define asthma; the first NPR record or the first asthma‐related prescription in the PDR was considered equal to the date of asthma diagnosis.

### 2.6 Statistical analysis

Follow‐up time started on the date of EoE diagnosis or index date for the reference group. Follow‐up ended with the date of asthma diagnosis, death, emigration, or December 31, 2019, whichever occurred first. Through Cox regression, we estimated hazard ratios (HRs) with 95% CIs for asthma in individuals with EoE. The concordance (which is the preferred goodness‐of‐fit measure for Cox proportional hazards regression) was 0.68 in our model. The interpretation is that 68% of outcomes are correctly classified by the model, which is considered satisfactory. We performed stratified analyses based on years of follow‐up (<1, 1 to <5, 5 to <10, and ≥10 years), age at first EoE diagnosis (childhood ≤17, 18 to ≤39, 40 to ≤59, and ≥60 years), sex (females vs. males), country of birth (Nordic vs. not Nordic), calendar year (year of biopsy and corresponding year in comparators: 1989–1999, 2000–2009, and 2010–2017), level of education (<9, 10–12, and ≥13 years), and autoimmune diseases (yes/no).

In a separate analysis, we compared the risk of asthma among individuals with EoE to their unaffected siblings. Only individuals with EoE (*n* = 781) and a full sibling (total number of siblings, *n* = 1304) were included in this analysis.

In a post hoc analysis, we also examined the risk of earlier asthma in individuals with EoE. We used conditional logistic regression after adjustment for the matching factors.

Statistics were performed using R statistical software (version 4.0.5, R Foundation for Statistical Computing) and the survival package (version 3.2, Therneau T, 2015: https://CRAN.Rproject.org/package=survival). HRs with 95% CIs that did not include 1.0 were considered statistically significant. In all analyses, risk estimates were adjusted for age, sex, calendar year, and county. In sibling analyses, the data were stratified by family and adjusted for the same variables as those used in the main analyses.

### 2.7 Ethical approval

The study was approved by the Ethics Review Board of Stockholm, Sweden (DNR 2014/1287‐31/4 and 2018/972‐32). The board waived informed consent as the study was strictly register‐based, which is the standard procedure in Sweden.^15^

## 3 RESULTS

A total of 1146 individuals with biopsy‐confirmed EoE from 1989 to 2017, 5022 matched reference individuals from the general population, and 1304 unaffected siblings of patients with EoE, all without a prior asthma diagnosis, were identified by linking the ESPRESSO cohort to the Swedish population and healthcare registers.

### 3.1 Background data

Baseline characteristics of the study population are presented in Table 1. The male to female predominance was 3:1. The median age at EoE‐diagnosis was 42.0 years (interquartile range [IQR] 24.0–56.0), while 13.2% were diagnosed in childhood (before the age of 18 years). Almost one third (29%) of individuals with EoE were between 18 and 40 years old at the start of follow‐up, and about 95% were from a Nordic country. The median follow‐up time for patients with EoE was 3.8 years (IQR 2.3–5.7), while 3.7% had a follow‐up time of more than 10 years. We found a similar level of education in EoE patients and reference individuals, while other autoimmune comorbidities were more likely to occur in EoE patients (5.9%) than in healthy comparators (2.3%) (Table 1).

**TABLE 1:** Summary statistics for EoE patients and reference individuals.

	EoE patients	Reference individuals
	*n* [%]	*n* [%]
Total	1146 [100.0]	5022 [100.0]
Male	858 [74.9]	3799 [75.6]
Female	288 [25.1]	1223 [24.4]
Years of follow‐up, yr		
Mean [SD]	4.4 [3.0]	4.8 [3.1]
Median [IQR]	3.8 [2.3–5.7]	4.2 [2.7–6.3]
<1	91 [7.9]	156 [3.1]
1 to <5	674 [58.8]	2983 [59.4]
5 to <10	339 [29.6]	1641 [32.7]
≥10 years	42 [3.7]	242 [4.8]
Age at the start of follow‐up, yr		
Mean [SD]	40.3 [20.4]	40.1 [20.0]
Median [IQR]	42.0 [24.0–56.0]	42.0 [24.0–55.0]
<18	151 [13.2]	636 [12.7]
18 to <40	332 [29.0]	1513 [30.1]
40 to <60	418 [36.5]	1851 [36.9]
≥60	245 [21.4]	1022 [20.4]
Year of start of follow‐up		
1989–1999	17 [1.5]	84 [1.7]
2000–2009	143 [12.5]	647 [12.9]
2010–2017	986 [86.0]	4291 [85.4]
Country of birth		
Nordic	1088 [94.9]	4161 [82.9]
Other	58 [5.1]	861 [17.1]
Education, yr		
Compulsory school, ≤9	187 [16.3]	960 [19.1]
Upper secondary school, 10–12	426 [37.2]	1878 [37.4]
College or university, ≥13	360 [31.4]	1359 [27.1]
Missing	173 [15.1]	825 [16.4]
Comorbidity		
Asthma (outcome)	140 [12.2]	174 [3.5]
Autoimmune disease^a^	68 [5.9]	117 [2.3]

Abbreviations: EoE, eosinophilic esophagitis; IQR, interquartile range; yr, years.

^a^ For a list of autoimmune diseases, see Supporting Information S1: Appendix.

### 3.2 Risk of asthma in individuals with EoE

Among individuals with EoE, 140 (12.2%) developed asthma during the 1989–2019 follow‐up, corresponding to an incidence rate of 28.1/1000 person‐years compared with 174 (3.5%) of reference individuals and an incidence rate of 7.2/1000 person‐years. This was equivalent to a 3.96‐fold increased risk of later asthma in EoE patients (95% CI = 3.16–4.96, *p* < 0.001) as presented in Table 2.

**TABLE 2:** Asthma incidence rates and adjusted HRs for EoE patients and reference individuals.

	EoE patients	Reference individuals	aHR [95% CI]
*N* total	1146	5022	
*N* asthma events	140	174	
Incidence proportion [%]	12.2	3.5	
Person years	4988	24,062	
Incidence rate/1000 py [95% CI]	28.1 [23.8–32.9]	7.2 [6.2–8.3]	3.96 [3.16–4.96]
Sex			
Males	25.1 [20.5–30.4]	6.5 [5.4–7.7]	3.86 [2.94–5.07]
Females	37.5 [28.0–49.2]	9.6 [7.4–12.3]	4.15 [2.77–6.21]
Years of follow‐up			
<1	66.7 [53.1–82.8]	5.4 [3.7–7.7]	12.39 [7.95–19.31]
1 < 5	16.5 [12.5–21.5]	8.0 [6.7–9.6]	2.09 [1.48–2.95]
5 < 10	19.0 [11.8–29.4]	6.0 [4.1–8.5]	3.00 [1.56–5.74]
≥10	20.4 [7.4–49.2]	10.7 [5.9–18.3]	2.94 [0.63–13.67]
Age at start of follow‐up			
<18 years	30.8 [22.0–42.2]	7.2 [5.3–9.7]	4.74 [2.93–7.67]
18–39 years	31.4 [23.1–41.8]	5.4 [3.9–7.3]	5.84 [3.68–9.29]
40–59 years	26.0 [19.4–34.1]	7.6 [6.0–9.7]	3.61 [2.44–5.34]
≥60 years	24.2 [16.0–35.3]	9.5 [6.9–12.7]	2.48 [1.45–4.24]
Year of start of follow‐up			
1989–1999	15.2 [6.2–33.4]	6.9 [3.7–12.0]	2.53 [0.60–10.64]
2000–2009	27.7 [19.5–38.2]	6.7 [4.9–9.0]	4.60 [2.82–7.48]
2010–2017	29.1 [24.1–34.9]	7.4 [6.2–8.8]	3.97 [3.06–5.16]
Country of birth			
Nordic	27.4 [23.1–32.3]	7.3 [6.2–8.5]	3.81 [2.99–4.84]
Other	42.5 [22.6–74.4]	6.9 [4.7–9.9]	8.06 [0.66–99.09]
Education, yr			
Compulsory school, ≤9	38.0 [26.4–53.3]	6.6 [4.6–9.1]	7.51 [3.54–15.96]
Upper secondary school, 10–12	28.7 [22.0–36.9]	8.3 [6.7–10.3]	3.67 [2.39–5.65]
College or university, ≥13	21.2 [15.1–29.2]	6.1 [4.4–8.2]	3.91 [2.05–7.45]
Missing	30.2 [20.8–42.6]	7.3 [5.1–10.0]	4.42 [2.57–7.61]
Comorbidity			
Autoimmunity	20.8 [9.7–40.4]	9.7 [4.3–19.9]	5.07 [1.08–23.66]
No autoimmunity	28.5 [24.1–33.5]	7.2 [6.2–8.3]	4.07 [3.24–5.11]

*Note*: aHRs, hazard ratios adjusted for age, sex, calendar year, country of birth, level of education, and concomitant autoimmune disease.

Abbreviations: CI, confidence interval; EoE, eosinophilic esophagitis; HR, hazard ratio; py, person‐years.

The highest risk of asthma was seen during the first year of follow‐up with an aHR of 12.39; 95% CI = 7.95–19.31. Between 1 and 10 years of follow‐up, the HR varied between 2 and 3 years, but beyond 10 years, EoE was no longer associated with asthma (Table 2).

The highest HRs for future asthma were seen in individuals diagnosed with EoE between 18 and 39 years (aHR 5.84; 95% CI = 3.68–9.29, *p* < 0.001), followed by those diagnosed in childhood (aHR 4.74; 95% CI = 2.93–7.67, *p* < 0.001) (Table 2). Female patients with EoE had a higher asthma incidence rate (37.5/1000 person‐years, 95% CI = 28.0–49.2) than their comparators (9.6/1000 person‐years, 95% CI = 7.4–12.3), while the difference was smaller in males (25.1/1000 vs. 6.5/1000 person‐years). Corresponding aHRs were 4.15 (95% CI = 2.77–6.21) in female and 3.86 (95% CI = 2.94–5.07) in male EoE patients, respectively (Table 2).

In stratified analyses, we used education as an indicator of socioeconomic status and found that the increased risk of asthma in EoE was independent of educational background (Table 2). Further, the occurrence of other autoimmune comorbidities among individuals with EoE did not change the risk estimates for asthma (aHR of 5.07 in those with other autoimmune conditions, and aHR = 4.07 in those without autoimmunity; Table 2 and Supporting Information S1: eTable 1). HRs for asthma were similar according to use of PPIs (3.32; 95% CI = 2.07–5.32 with PPIs vs. 4.37; 95% CI = 3.32–5.76 without, *p* for heterogeneity 0.32) (Table S2).

### 3.3 Sibling comparisons

We identified 1304 unaffected siblings in 781 patients with EoE (Table 3). Of these, 46 (3.5%) developed asthma during follow‐up, corresponding to an incidence rate of 7.1 per 1000 person‐years. Compared to their siblings without EoE, individuals with EoE were at a greater risk of asthma with an aHR of 4.97 (95% CI = 3.13–7.92, *p* < 0.001) (Table 4). Siblings were not matched; the proportion of male siblings was 51.6% compared with 75.3% in the EoE cohort (Table 3). However, this should not be an issue as HRs were adjusted for sex. The occurrence of other autoimmune comorbidities was similar among individuals with EoE (5.2%) and their unaffected siblings (4.9%) (Table 3 and Supporting Information S1: eTable 2).

**TABLE 3:** Summary statistics for EoE patients and their unaffected siblings.

	EoE patients	Siblings
	*n* [%]	*n* [%]
Total	781 [100.0]	1304 [100.0]
Male	588 [75.3]	673 [51.6]
Female	193 [24.7]	631 [48.4]
Years of follow‐up, yr		
Mean [SD]	4.4 [3.1]	5.0 [3.3]
Median [IQR]	3.9 [2.5–5.7]	4.3 [2.9–6.3]
<1	59 [7.6]	38 [2.9]
1 < 5	458 [58.6]	749 [57.4]
≥5	264 [33.8]	517 [39.6]
Age at start of the follow‐up, yr		
Mean [SD]	38.8 [18.7]	40.4 [19.3]
Median [IQR]	41.0 [23.0–53.0]	42.0 [26.0–55.0]
<18	106 [13.6]	119 [9.1]
18 < 40	233 [29.8]	392 [30.1]
40 < 60	311 [39.8]	523 [40.1]
≥60	131 [16.8]	270 [20.7]
Year of start of follow‐up		
1989–1999	10 [1.3]	21 [1.6]
2000–2009	92 [11.8]	154 [11.8]
2010–2017	679 [86.9]	1129 [86.6]
Country of birth		
Nordic	763 [97.7]	1271 [97.5]
Other	18 [2.3]	33 [2.5]
Education, yr		
Compulsory school, ≤9	111 [14.2]	203 [15.6]
Upper secondary school, 10–12	287 [36.7]	486 [37.3]
College or university, ≥13	262 [33.5]	432 [33.1]
Missing	121 [15.5]	183 [14.0]
Comorbidity		
Asthma	94 [12.0]	46 [3.5]
Autoimmunity	41 [5.2]	64 [4.9]

*Note*: aHRs, hazard ratios adjusted for age, sex, calendar year, country of birth, level of education, and concomitant autoimmune disease.

Abbreviations: EoE, eosinophilic esophagitis; IQR, interquartile range; yr, years.

**TABLE 4:** Asthma incidence rates and adjusted HRs for EoE patients and their unaffected siblings.

	EoE patients	Siblings	aHR [95% CI]
*N* total	781	1304	
*N* events	94	46	
Incidence proportion [%]	12.0	3.5	
Person years	3461	6455	
Incidence rate/1000 py [95% CI]	27.2 [22.2–32.9]	7.1 [5.3–9.3]	4.97 [3.13–7.92]
Sex			
Males	25.6 [20.2–32.0]	5.0 [3.2–7.7]	4.58 [2.18–9.62]
Females	32.3 [22.1–45.8]	9.4 [6.6–13.1]	5.57 [1.84–16.88]
Years of follow‐up			
<1	61.4 [46.1–80.4]	9.3 [5.3–15.2]	16.53 [4.71–58.06]
1 < 5	17.3 [12.5–23.5]	7.8 [5.5–10.9]	3.06 [1.61–5.85]
≥5	18.9 [11.1–30.4]	3.4 [1.5–7.0]	5.93 [1.67–21.02]
Age at start of follow‐up			
<18	30.9 [20.8–44.4]	9.5 [5.4–15.9]	2.57 [0.96–6.83]
18–39	24.8 [16.6–35.9]	4.3 [2.2–7.7]	[NA]
40–59	26.8 [19.3–36.4]	7.2 [4.6–10.9]	4.07 [1.91–8.65]
≥60	26.6 [15.3–43.6]	9.6 [5.1–16.8]	[NA]
Year of start of follow‐up			
1989–1999	11.1 [3.4–30.8]	4.8 [1.5–13.3]	[NA]
2000–2009	24.0 [15.2–36.2]	7.0 [3.9–12.0]	3.25 [1.27–8.34]
2010–2017	29.3 [23.3–36.3]	7.4 [5.3–10.0]	6.18 [3.44–11.12]
Country of birth			
Nordic	27.1 [22.1–32.9]	6.8 [5.1–9.0]	5.12 [3.19–8.21]
Other	29.6 [9.1–82.4]	21.8 [7.9–52.6]	[NA]
Education, yr			
Compulsory school, ≤9	32.2 [19.6–50.4]	4.0 [1.6–8.8]	
Upper secondary school, 10–12	31.6 [23.1–42.2]	6.3 [3.8–9.9]	5.87 [1.89–18.25]
College or university, ≥13	19.8 [13.1–28.9]	7.6 [4.7–11.8]	7.64 [1.53–38.16]
Missing	27.8 [17.6–42.0]	11.3 [6.4–18.9]	2.05 [0.75–5.58]
Comorbidity			
Autoimmunity	30.5 [13.4–62.5]	10.6 [3.9–25.6]	[NA]
No autoimmunity	27.0 [22.0–32.9]	7.0 [5.2–9.2]	4.97 [3.06–8.05]

*Note*: aHRs, hazard ratios adjusted for age, sex, calendar year, country of birth, level of education, and concomitant autoimmune disease.

Abbreviations: CI, confidence interval; EoE, eosinophilic esophagitis; HR, hazard ratio; NA, not applicable due to low number of cases; py, person‐years.

### 3.4 Earlier asthma and EoE

In a separate analysis, we assessed the risk of earlier asthma in EoE patients. We identified 1659 individuals with biopsy‐verified EoE in the ESPRESSO cohort. These were matched to 8171 non‐EoE reference individuals from the general population (1:5 ratio). Some 513/1659 (30.9%) EoE patients and 1462/8171 (17.9%) reference individuals had an earlier asthma diagnosis. Through conditional logistic regression analysis, we found an OR of 3.49; 95% CI = 3.07–3.96 after adjustment for the matching factors (the same as in main analyses).

## 4 DISCUSSION

In this nationwide population‐based cohort study of 1146 individuals with biopsy‐verified EoE over 28 years, we found a statistically significantly increased risk of asthma in EoE compared with reference individuals from the general population and unaffected siblings.

### 4.1 Main findings discussed in comparison with earlier research

After adjusting for sex, age, country of birth, calendar year, level of education, and auto‐immune diseases we found a 4‐fold increased risk of new‐onset asthma in individuals with EoE compared with the general population. Our results confirm earlier findings of a positive association between the two diseases.^2^, ^3^, ^4^, ^5^, ^16^, ^17^, ^18^, ^19^, ^20^, ^21^, ^22^, ^23^, ^24^, ^25^ However, according to a 2017 systematic review and meta‐analysis of 21 case‐control studies, the quality of the available evidence on the risk of atopic manifestations (including asthma) among patients with EoE is still only moderate with many studies being retrospective in design, while only few have used stringent diagnostic criteria to clearly define asthma (usually requiring a medical diagnosis by a physician).^4^ Earlier in 2005, Simon et al. conducted a study on 31 individuals with EoE and reported high frequencies of concomitant atopic diseases (68%), sensitization to inhaled allergens (77%), and to food allergens (>50%). The authors proposed that allergic airway disease often precedes EoE, and that EoE should be considered as an additional manifestation of atopy. They hypothesized that initial allergen sensitization might take place in the airways and suggested the extension of the united airways concept to the upper gastrointestinal tract and the esophagus.^26^ Our study found a 4‐fold increased risk of later asthma in individuals with EoE compared with the general population, suggesting that the pathophysiologic mechanisms behind the EoE‐asthma relationship should be further investigated.

In 2016, Krupp et al. suggested that particularly in children with EoE, the risk of asthma may have been underestimated,^16^ while in 2018, Hill et al. also discussed EoE as a possible component of the atopic march.^3^ Yet, it is notable that EoE can occur beyond childhood, and in otherwise non‐atopic adults,^3^ implying that a causal relationship between the two conditions cannot be established.^4^ In our study, we observed a high incidence of asthma in childhood and young adulthood onset EoE. The highest HRs for asthma were seen in individuals diagnosed with EoE aged 18–39 years; but the HR was also high in childhood onset EoE. The risk of asthma was particularly elevated in the first year of follow‐up, possibly because of more intense surveillance just after EoE diagnosis or due to shared pathophysiologic mechanisms and inflammation triggering both EoE and asthma. Beyond 10 years of follow‐up, the aHR of asthma was no longer statistically significant probably due to insufficient power (only 3.7% of EoE patients had a follow‐up time of ≥10 years). Intriguingly, the OR of earlier asthma was 3.49 in individuals with EoE, implying that there might be a bidirectional association between the two conditions.

There is some evidence that EoE might be associated with an increased risk of autoimmune disorders.^27^, ^28^ In 2017, a US study reported a positive association between EoE and celiac disease, Crohn's disease, ulcerative colitis, and Hashimoto's thyroiditis; the authors encouraged physicians to be aware of these comorbid conditions.^28^ Nonetheless, in our study, the occurrence of other autoimmune comorbidities among individuals with EoE did not change the relative risk of asthma significantly.

### 4.2 Strengths and limitations

Among this study's strengths is the large sample size including 1146 individuals with EoE with a 3:1 male to female predominance, in line with previous reports.^1^, ^29^, ^30^ The large sample size provided accuracy in risk estimates and allowed for stratified analyses. In particular, asthma has been associated with low socioeconomic status and education.^31^ To examine whether education was an independent risk factor for asthma, we performed stratification by educational level in patients with EoE. The positive association between EoE and asthma was persistent across educational strata. Similarly, we found no significant difference in HRs for asthma according to the use of PPIs in EoE individuals. In addition, through the TPR, we collected data on emigration and mortality and were able to calculate precise follow‐up times. Considering that national healthcare registers in Sweden have virtually complete coverage and follow‐up, and that the public healthcare system is tax‐funded,^32^ with access independent of income or education, loss to follow‐up or bias due to socioeconomic status should be minimal in this study.

We used Morphology/SnoMed code T62 (esophagus) with M47150 (eosinophilia) for our EoE diagnosis. A previous validation of the SnoMed code for EoE diagnosis in the ESPRESSO cohort found a PPV of 89%, reflecting true EoE disease according to patient chart data.^14^ The validation was in accordance with the 2018 guidelines' diagnostic criteria.^33^ Nevertheless, we cannot rule out that some mild or evolving cases of EoE may have been missed. The high validity of our case definition for EoE diagnosis along with the population‐based design should have minimized selection bias. Moreover, the SnoMed code for EoE was introduced much earlier in Sweden than the relevant ICD‐code (year 2012), and using the SnoMed code we could start our study already in 1989, allowing for a longer follow‐up and higher statistical power than if we had used the ICD code for exposure definition.

Similarly, we used register‐based data to define asthma. Unfortunately, we lacked data on asthma medication before 2005, while outpatient data were first added in the NPR in 2001. However, a 2013 study by Ortqvist et al. reported that the quality of physician‐diagnosed asthma in the Swedish NPR is high, while the use of asthma medication according to the PDR had a PPV of 89% for asthma in both school‐age children and adults.^34^ The relatively high PPV implies that asthma medication reported in the PDR can be used as a proxy for asthma diagnosis.^34^ Further, we performed a sensitivity analysis restricted to post 2005 data, resulting in an aHR for asthma of 4.03; 95% CI: 3.18–5.18 in EoE patients (see Supporting Information S1: Appendix), very similar to the aHR of 3.96 reported in our main analysis, since only 36 EoE patients had a biopsy prior to 2006. All in all, the high validity of both exposure and outcome supports our study's generalizability. We do, however, acknowledge that the use of dispensed prescriptions of asthma medications to define asthma has its limitations since swallowed ICS are also commonly used for EoE treatment,^35^ which might have somewhat influenced the risk of asthma. Also, considering that most EoE‐patients originated from the Nordic countries, our results may not widely apply to non‐Western populations.

Although early environmental exposures such as infection and antibiotic use have been associated with increased risk of EoE,^36^ additional individual‐specific factors may contribute. Our sibling analyses are another strength of this study, allowing us to adjust for unmeasured genetic, lifestyle, and many early environmental factors such as diet and air quality. As residual confounding is an obvious risk in all observational research, the use of unaffected sibling comparators minimized intrafamilial confounding. Intriguingly, compared with their siblings, individuals with EoE were at an almost 5‐fold greater risk of asthma, suggesting that shared environmental and genetic components are unlikely to explain the observed positive association. Our findings instead imply a direct linkage between EoE and asthma comorbidity. Neither does the association seem to be a result of an altered health seeking behavior following EoE diagnosis, as this change would possibly have affected siblings as well.

The major limitation of our study is the lack of data regarding other allergic manifestations or gastroesophageal reflux disease (GERD), co‐existing at the time of EoE diagnosis, that might have affected the risk of asthma.^37^ EoE and GERD frequently overlap, while GERD is believed to affect asthma development through microaspiration, airway hyperresponsiveness, and both local and central reflexes including elevated vagal tone.^38^ Similar mechanisms might exist for the association of EoE with asthma considering the pulmonary‐esophageal relationship in airway disease and similarities between GERD and EoE.

Further, as we did not have information on EoE disease activity or severity, we cannot rule out that the positive association with asthma may primarily apply to moderate‐to‐severe EoE. EoE is a rather new diagnosis and, hence, most patients were diagnosed recently with a median follow‐up time of 3.8 years, while only 3.7% had a follow‐up time beyond 10 years. In addition, we noted a relatively high median age (42 years) of EoE patients at the start of follow‐up. Although wheezing is considered the hallmark of asthma in early childhood, which is well before the median age of EoE diagnosis in our study, the majority of asthma has its onset later in adulthood,^37^ while asthma diagnosis becomes more accurate with increasing age.^34^ On the other hand, the proportion of allergic asthma decreases with advancing age at onset.^37^ In the current study, we did not have the statistical power to examine subgroups of asthma phenotypes.

### 4.3 Clinical implications and future perspectives

This nationwide population‐based study suggests that individuals with EoE have an increased risk of asthma compared with the general population and their siblings. Our findings encourage healthcare professionals to assess asthma comorbidity in patients with EoE and consider a thorough evaluation by an asthma specialist, supporting current clinical recommendations.^2^ More research on comorbidity is needed to improve the overall health care of patients with EoE. Future studies should focus on examining whether the observed association with asthma is restricted to more severe EoE disease.

## AUTHOR CONTRIBUTIONS

**Niki Mitselou**: Conceptualization; writing—original draft; methodology; funding acquisition; writing—review and editing. **Amiko Uchida**: Methodology; funding acquisition; writing—review and editing; conceptualization. **Bjorn Roelstraete**: Methodology; data curation; formal analysis. **Erik Melén**: Supervision; writing—review and editing. **John J. Garber**: Writing—review and editing. **Jonas F. Ludvigsson**: Supervision; conceptualization; methodology; writing—review and editing; data curation; formal analysis.

## CONFLICT OF INTEREST STATEMENT

Dr. Uchida consults for Sanofi‐Regeneron and AstraZeneca. Dr. Ludvigsson coordinates a study on behalf of the Swedish IBD quality register (SWIBREG), which has received funding from Janssen corporation. Dr. Ludvigsson has also received financial support from MSD in developing a paper reviewing national healthcare registers in China. Dr. Ludvigsson is currently discussing potential research collaboration with Takeda. The other authors have no conflicts of interest to disclose.

## Supporting information

Supporting Information S1

Tables S1–S2

## Data Availability

The data that support the findings of this study are available on request from the corresponding author. The data are not publicly available due to privacy or ethical restrictions.
