# Supplementary material for: Risk of asthma in individuals with eosinophilic esophagitis: Population‐based cohort study with sibling analyses
Source: Clin Transl Allergy. 2025 May 31;15(6):e70068. doi: 10.1002/clt2.70068 (PMC12126120; doi:10.1002/clt2.70068)
Supplement: Supplementary file 1 — Supporting Information S1 [file CLT2-15-e70068-s001.docx]

| **eTable 1. Autoimmune diseases in EoE patients and reference individuals** | | |
| --- | --- | --- |
|  | EoE patients | Reference individuals |
|  | n [%] | n [%] |
| **Autoimmune disease** | 68 [5.9] | 117 [2.3] |
| **-Celiac** | 32 [2.8] | 1 [0.0] |
| **-Diabetes** | 4 [0.3] | 13 [0.3] |
| **-Psoriasis** | 16 [1.4] | 61 [1.2] |
| **-Systemic lupus erythematosus (SLE)** | 1 [0.1] | 2 [0.0] |
| **-Hypothyroid disease** | 2 [0.2] | 2 [0.0] |
| **-Hyperthyroid disease** | 4 [0.3] | 15 [0.3] |
| **-Sarcoidosis** | 5 [0.4] | 7 [0.1] |
| **-Primary biliary cirrhosis** | 1 [0.1] | 0 [0.0] |
| **-ANCA** | 1 [0.1] | 4 [0.1] |
| **-Pelvo (ankylosing spondylitis)** | 2 [0.2] | 9 [0.2] |
| **-Autoimmune hepatitis** | 3 [0.3] | 4 [0.1] |
| **-Primary sclerosing cholangitis** | 0 [0.0] | 0 [0.0] |

| **eTable 2. Autoimmune diseases in EoE patients and their unaffected siblings** | | |
| --- | --- | --- |
|  | EoE patients | Siblings |
|  | n [%] | n [%] |
| **Autoimmune disease** | 41 [5.2] | 64 [4.9] |
| **-Celiac** | 23 [2.9] | 16 [1.2] |
| **-Diabetes** | 2 [0.3] | 8 [0.6] |
| **-Psoriasis** | 7 [0.9] | 22 [1.7] |
| **-Systemic lupus erythematosus (SLE)** | 1 [0.1] | 0 [0.0] |
| **-Hypothyroid disease** | 1 [0.1] | 4 [0.3] |
| **-Hyperthyroid disease** | 2 [0.3] | 7 [0.5] |
| **-Sarcoidosis** | 3 [0.4] | 2 [0.2] |
| **-Primary biliary cirrhosis** | 0 [0.0] | 0 [0.0] |
| **-ANCA** | 1 [0.1] | 2 [0.2] |
| **-Pelvo (ankylosing spondylitis)** | 1 [0.1] | 4 [0.3] |
| **-Autoimmune hepatitis** | 2 [0.3] | 1 [0.1] |
| **-Primary sclerosing cholangitis** | 0 [0.0] | 2 [0.2] |

| **eTable 3. Sensitivity analysis (post 2005 data): Summary statistics for EoE patients and general population controls** | | | | | |
| --- | --- | --- | --- | --- | --- |
|  | | | | | |
|  | | Exposed | | Population controls | |
|  | | | | | |
|  | | n [%] | | n [%] | |
| Total | | 1110 [100.0] | | 4849 [100.0] | |
| Male | | 837 [75.4] | | 3698 [76.3] | |
| Female | | 273 [24.6] | | 1151 [23.7] | |
| YEARS OF FOLLOW UP | |  | |  | |
| Mean [SD] years | | 4.1 [2.5] | | 4.5 [2.4] | |
| Median [IQR] years | | 3.7 [2.3-5.6] | | 4.1 [2.7-5.9] | |
| < 1 years | | 88 [7.9] | | 152 [3.1] | |
| 1 < 5 years | | 668 [60.2] | | 2966 [61.2] | |
| 5 < 10 years | | 333 [30.0] | | 1624 [33.5] | |
| > = 10 years | | 21 [1.9] | | 107 [2.2] | |
| AGE AT START FOLLOW UP | |  | |  | |
| Mean [SD] years | | 40.3 [20.2] | | 40.1 [19.8] | |
| Median [IQR] years | | 42.0 [24.0-56.0] | | 42.0 [25.0-55.0] | |
| < 18 years | | 143 [12.9] | | 599 [12.4] | |
| 18 < 40 years | | 321 [28.9] | | 1457 [30.0] | |
| 40 < 60 years | | 411 [37.0] | | 1820 [37.5] | |
| > = 60 years | | 235 [21.2] | | 973 [20.1] | |
| YEAR OF START FOLLOW UP | |  | |  | |
| 2006 - 2010 | | 201 [18.1] | | 900 [18.6] | |
| 2011 - 2017 | | 909 [81.9] | | 3949 [81.4] | |
| COUNTRY OF BIRTH | |  | |  | |
| Nordic | | 1053 [94.9] | | 4006 [82.6] | |
| Other | | 57 [5.1] | | 843 [17.4] | |
| NA | | 0 [0.0] | | 0 [0.0] | |
| EDUCATION | |  | |  | |
| Compulsory school, < = 9 yrs | | 178 [16.0] | | 909 [18.7] | |
| Upper secondary school (10-12 yrs) | | 419 [37.7] | | 1830 [37.7] | |
| College or university (> = 13 yrs) | | 350 [31.5] | | 1334 [27.5] | |
| NA1 | | 163 [14.7] | | 776 [16.0] | |
| COMORBIDITY | |  | |  | |
| Asthma | | 128 [11.5] | | 152 [3.1] | |
| autoimmun | | 67 [6.0] | | 116 [2.4] | |
| celiac | | 31 [2.8] | | 1 [0.0] | |
| diab | | 4 [0.4] | | 13 [0.3] | |
| Psoriasis | | 16 [1.4] | | 61 [1.3] | |
| SLE | | 1 [0.1] | | 2 [0.0] | |
| thyroid | | 2 [0.2] | | 2 [0.0] | |
| hyperthyroid | | 4 [0.4] | | 14 [0.3] | |
| Sarcoidosis | | 5 [0.5] | | 7 [0.1] | |
| pbc | | 1 [0.1] | | 0 [0.0] | |
| ANCA | | 1 [0.1] | | 4 [0.1] | |
| Pelvo | | 2 [0.2] | | 9 [0.2] | |
| hepatitis | | 3 [0.3] | | 4 [0.1] | |
| cholangitis | | 0 [0.0] | | 0 [0.0] | |
|  | | | | | |
| **eTable 4. Sensitivity analysis (post 2005 data): Asthma incidence rates for EoE patients and general population controls** | | | | | |
|  | | | | | |
|  | | Exposed | | Population controls | |
|  | | | | | |
| N Total | | 1110 | | 4849 | |
| N events | | 128 | | 152 | |
| Incidence proportion [%] | | 11.5 | | 3.1 | |
| Person years | | 4587 | | 21689 | |
| Incidence rate/1000 p-years [95% CI] | | 27.9 [23.5-32.9] | | 7.0 [6.0-8.2] | |
| SEX | |  | |  | |
| Males | | 25.2 [20.5-30.6] | | 6.4 [5.3-7.6] | |
| Females | | 37.2 [27.2-49.7] | | 9.1 [6.8-12.0] | |
| YEARS OF FOLLOW UP | |  | |  | |
| < 1 | | 66.0 [52.3-82.3] | | 5.4 [3.7-7.7] | |
| 1 < 5 | | 15.8 [11.8-20.8] | | 8.0 [6.6-9.6] | |
| 5 < 10 | | 19.5 [11.7-30.9] | | 5.8 [3.8-8.5] | |
| > = 10 | | 0.0 [0.0-0.0] | | 0.0 [0.0-0.0] | |
| AGE AT START FOLLOW UP | |  | |  | |
| < 18 years | | 30.9 [21.5-43.1] | | 7.8 [5.6-10.6] | |
| 18 - 39 years | | 31.0 [22.6-41.8] | | 4.4 [3.0-6.2] | |
| 40 - 59 years | | 26.1 [19.4-34.4] | | 7.7 [6.0-9.8] | |
| > = 60 years | | 23.6 [15.2-35.3] | | 9.0 [6.4-12.4] | |
| YEAR OF START FOLLOW UP | |  | |  | |
| 2006 - 2010 | | 29.7 [22.1-39.2] | | 6.7 [5.1-8.8] | |
| 2011 - 2017 | | 27.1 [21.9-33.1] | | 7.1 [5.9-8.6] | |
| COUNTRY | |  | |  | |
| Nordic | | 27.4 [22.9-32.5] | | 7.0 [5.9-8.2] | |
| Non-Nordic | | 37.8 [19.4-68.1] | | 7.2 [4.8-10.3] | |
| EDUCATION | |  | |  | |
| Compulsory school (< = 9 yrs) | | 34.9 [23.3-50.5] | | 5.6 [3.8-8.2] | |
| Upper secondary school (10-12 yrs) | | 29.0 [22.1-37.5] | | 8.2 [6.5-10.3] | |
| College or university (> = 13 yrs) | | 20.6 [14.4-28.8] | | 5.6 [4.0-7.7] | |
| NA | | 32.8 [22.3-46.8] | | 8.0 [5.6-11.1] | |
| COMORBIDITY | |  | |  | |
| Autoimmunity | | 21.70 [10.18-42.20] | | 7.87 [3.20-17.26] | |
| No autoimmunity | | 28.30 [23.72-33.54] | | 6.99 [5.95-8.16] | |
|  | | | | | |

| **eTable 5. Sensitivity analysis (post 2005 data): Asthma hazard ratios for EoE patients versus general population controls** | | |
| --- | --- | --- |
|  | | |
|  | Exposed | Population controls |
|  | | |
|  | n/1000pyrs | HR [95% CI] |
| Total |  | 4.03 [3.18-5.11] |
| SEX |  |  |
| Males |  | 3.93 [2.96-5.22] |
| Females |  | 4.17 [2.71-6.43] |
| YEARS OF FOLLOW UP |  |  |
| < 1 |  | 12.22 [7.78-19.21] |
| 1 < 5 |  | 2.00 [1.40-2.86] |
| 5 < 10 |  | 3.13 [1.56-6.27] |
| > = 10 |  | NA [NA-NA] |
| AGE AT START FOLLOW UP |  |  |
| < 18 years |  | 4.38 [2.64-7.26] |
| 18 - 39 years |  | 7.04 [4.25-11.65] |
| 40 - 59 years |  | 3.55 [2.38-5.29] |
| > = 60 years |  | 2.54 [1.43-4.50] |
| YEAR OF START FOLLOW UP |  |  |
| 2006 - 2010 |  | 4.48 [2.97-6.78] |
| 2011 - 2017 |  | 3.86 [2.89-5.16] |
| COUNTRY |  |  |
| Nordic |  | 3.94 [3.06-5.08] |
| Non-Nordic |  | 8.06 [0.66-99.09] |
| EDUCATION |  |  |
| Compulsory school (< = 9 yrs) |  | 9.18 [3.77-22.34] |
| Upper secondary school (10-12 yrs) |  | 3.79 [2.43-5.91] |
| College or university (> = 13 yrs) |  | 3.71 [1.91-7.21] |
| NA |  | 4.37 [2.49-7.69] |
| COMORBIDITY |  |  |
| Autoimmunity |  | 5.35 [1.15-24.92] |
| No autoimmunity |  | 4.11 [3.23-5.23] |
|  | | |
