# Supplementary material for: Risk of asthma in individuals with eosinophilic esophagitis: Population‐based cohort study with sibling analyses
Source: Clin Transl Allergy. 2025 May 31;15(6):e70068. doi: 10.1002/clt2.70068 (PMC12126120; doi:10.1002/clt2.70068)
Supplement: Supplementary file 2 — Tables S1–S2 [file CLT2-15-e70068-s002.docx]

**Supplementary Table 1. ATC codes for PPIs**

| **PPI** | **ATC code** |
| --- | --- |
| omeprazole | A02BC01 |
| pantoprazole | A02BC02 |
| lansoprazole | A02BC03 |
| rabeprazole | A02BC04 |
| esomeprazole | A02BC05 |
| dexlansoprazole | A02BC06 |
| dexrabeprazole | A02BC07 |

ATC, Anatomic Therapeutic Chemical; PPI, proton pump inhibitor.

**Supplementary Table 2. HRs for asthma in EoE patients by PPI use.**

| **PPI medication** | **aHR* [95%CI]** | **N/cases** | **P heterogeneity** |
| --- | --- | --- | --- |
| **PPI** | 3.32 [2.07-5.32] | 30/360 | 0.32 |
| **No PPI** | 4.37 [3.32-5.76] | 98/750 |  |

HR, hazard ratio; EoE, eosinophilic esophagitis; PPI, proton pump inhibitor; CI, confidence interval.

aHRs, hazard ratios adjusted for age, sex, calendar year, country of birth, level of education, and concomitant autoimmune disease.
